# Defect Chemistry and Chemical Looping Performance of La_1−x_M_x_MnO_3_ (M = Sr, Ca, (x = 0–0.5)) Perovskites

**DOI:** 10.3390/nano12193461

**Published:** 2022-10-03

**Authors:** Antigoni Evdou, Theofilos Georgitsis, Charitini Matsouka, Eleni Pachatouridou, Eleni Iliopoulou, Vassilios Zaspalis

**Affiliations:** 1 Laboratory of Inorganic Materials, Laboratory of Environmental Fuels & Hydrocarbons, Chemical Process and Energy Resources Institute, Center for Research and Technology Hellas, 57001 Thessaloniki, Greece; 2 Laboratory of Materials Technology, Faculty of Chemical Engineering, School of Engineering, Aristotle University of Thessaloniki, 54124 Thessaloniki, Greece

**Keywords:** perovskites, defect chemistry, chemical looping, Oxygen Transfer Capacity (OTC)

## Abstract

La_1−x_M_x_MnO_3_ (M = Sr, Ca, (x = 0–0.5)) materials of the perovskite structure are synthesized by a co-precipitation method. They are subsequently investigated for their performance in a chemical looping process (fuel CH_4_) using thermogravimetric analysis with simultaneous reaction. The goal of this work is to determine the relation between the defect chemistry of the materials and their behavior in chemical looping processes. A defect model is proposed that provides an explanation of the dependency of the Oxygen Transfer Capacity and of the CO_2_/CO selectivity on composition. It appeared that the fuel may react with various types of oxygen available within the materials, generated by different mechanisms. The relative amounts of each oxygen type determine the CO_2_/CO selectivity and depend on the material composition as well as on the partial pressure of oxygen used for regenerating the materials.

## 1. Introduction

Chemical looping (CL) is a general term that refers to a concept according to which a chemical reaction is decomposed into two subreactions facilitated by the solid oxygen carrier (OC). During the first step of this cyclic process, the OC is oxidized by taking up oxygen from an oxygen source. It is subsequently used for the oxidation of the fuel during the second step. The reduced or anion-deficient OC is then again subjected to oxidation and the cycle is repeated [1,2,3]. According to the differences in the oxygen source (i.e., air and/or H_2_O) or the target gas products of the reaction (i.e., CO_2_, syngas and/or H_2_), many processes based on chemical looping technology have been defined, among which include chemical looping combustion (CLC), chemical looping reforming (CLR), chemical looping hydrogen generation (CLHG) or chemical looping gasification (CLG) [4]. Perhaps the most extensively studied is the CLC process since it has been viewed as a promising technology to capture CO_2_ from fossil fuel combustion for power and heat generation [2,3]. However, CLR is also an interesting and extensively studied CL process since it enables the efficient and environmentally friendly production of syngas and/or hydrogen that may be further used for the production of chemicals [5,6]. CLHG has also attracted a lot of research interest and is considered as competing with other hydrogen production technologies under development [7,8]. Depending on the mechanism by which oxygen reacts with the fuel towards total oxidation, a subcategory of the CLC process is the chemical looping with oxygen uncoupling (CLOU) process. Both processes lead to total oxidation with the major difference that, unlike the CLC process, in the CLOU process, the OC releases gas phase oxygen in the fuel reactor, which reacts with the fuel in the gas phase [9].

A very important roadblock towards implementation of any CL technology is the OC. The criteria that need to be fulfilled include thermodynamic or mechanical stability, redox activity and selectivity towards desired products or low cost. An important criterion for a material suitability for a CL process is the Oxygen Transfer Capacity (OTC) which is usually defined as the amount of oxygen (in wt.%) which the OC can deliver to the fuel per cycle. A great variety of materials has been investigated in relation to certain CL processes, including pure transition metal oxides or mixed oxides either as active materials or semiactive supports [10].

Perovskites consist also of a material category that has attracted a lot of interest as OC materials in CL, in particular in CLR, processes [10,11,12,13,14]. Although the OTC of perovskite materials is much lower than this exhibited by transition metal oxides [10,15], there are other advantages, such as high cyclic redox stability, superior oxygen mobility or the ability to simultaneously accommodate several different cations in their lattice and to exhibit various degrees of oxygen excess or deficiency, that make them very interesting materials in CLR processes.

For perovskites, it is well-known that their particular point defect chemistry lies on the basis for their macroscopic oxygen evolution, diffusion coefficient, electric or magnetic behavior and their dependencies on temperature or oxygen partial pressure [16,17,18]. It is therefore quite logic to assume that there is also a relation between the defect chemistry and the performance of perovskites in chemical looping processes that could explain the effect of aliovalent substitutions on the OTC and on the reaction product selectivity. Such a relation is not clear in the literature, although macroscopic chemical looping performance is well-described for a wide variety of materials.

Perovskites derived from LaMnO_3±δ_ by substitution of alkaline-earth metal for La are the preferred materials for several applications at moderate and high temperatures, such as solid oxide fuel cells and mixed ionic and electronic conducting membranes, due to their ability to meet appropriate requirements such as high melting point, large electronic and oxygen ion conductivity and chemical or mechanical stability in both oxidizing and reducing environments [19,20]. These properties make the materials very interesting OC in CL or other chemical processes, as evidenced by the significant research interest they have attracted [21,22,23].

In this paper, La_1−x_Sr_x_MnO_3±δ_ (x = 0, 0.1, 0.3, 0.5) and La_1−x_Ca_x_MnO_3±δ_ (x = 0, 0.3, 0.5) perovskites are synthesized by a co-precipitation method and subsequently examined under simultaneous thermogravimetric and chemical looping reaction conditions, using CH_4_ as fuel. The purpose of this work is to provide a defect chemistry model that describes their behavior in chemical looping processes.

## 2. Materials and Methods

Perovskite materials in powder form are synthesized by the co-precipitation method [24]. The cation precursors used are La(NO_3_)_3_⦁6H_2_O (Alfa Aesar, Haverhill, MA, USA, 99.9%), Mn(NO_3_)_2_⦁4H_2_O (Sigma Aldrich, St. Louis, MI, USA, 99.9%), Sr(NO_3_)_2_ (Sigma Aldrich, 98%) and Ca(NO_3_)_2_⦁4H_2_O (Sigma Aldrich, 99%) while NaOH (VWR Chemicals, Radnor, PA, USA, 98.9%) is the precipitation agent. The appropriate amounts of each nitrate salt, corresponding to 30 g of final product, are diluted in 40 mL doubly ionized water while a 2M aqueous solution is prepared by adding 600 mL doubly ionized water to the precipitation agent. The nitrate salt solutions are initially mixed together and subsequently added to the precipitating agent solution. The final solution is left under magnetic stirring at room temperature and at pH = 12–13 for 40 min and subsequently heated at 90 °C for 4 h. The already formed precipitate is filtered, washed several times in order to remove possible NaNO_3_ residues, dried, de-agglomerated and finally heated at 1000 °C for 6 h in air. The final products are La_1−x_Sr_x_MnO_3_ or La_1−x_Ca_x_MnO_3_ (x = 0–0.5) perovskites.

The structure and phases of the synthesized powders are determined by X-ray diffraction (XRD) with CuKa radiation (Siemens D500, München, Germany). Morphological examination is performed with a JEOL 6300 scanning electron microscope.

**Figure 1:**
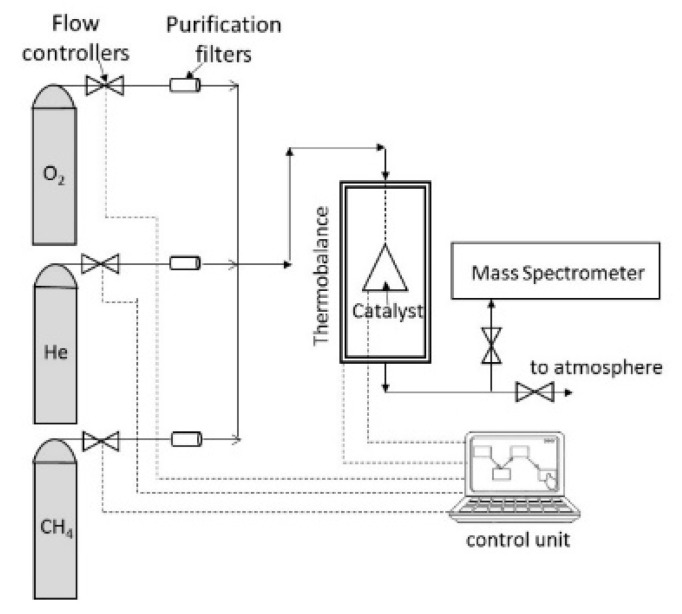
Schematic diagram of the experimental setup used for the reaction-thermogravimetric experiments.

Simultaneous reaction and thermogravimetric experiments are performed in a multicomponent thermogravimetric analyzer with chemical analysis of the exit stream by mass spectrometry (IGA, Hiden-Isochema, Warrington, England), shown in Figure 1. During all experiments, the total pressure is constantly maintained at 105 kPa. An accurately pre-weighted amount of material (~70–100 mg) is placed in the thermobalance and heated under helium atmosphere at a constant temperature, where enough time is left in order to equilibrate. Subsequently, either oxygen is introduced at various partial pressures or CH_4_. The exact atmosphere sequence will be mentioned in the appropriate result section. The material weight changes as well as the intensities of the mass spectrometer peaks at selected Atomic Mass Units (AMU) are being monitored.

For the calculation of the relative weight loss or gains, the specimen weight in equilibrium with helium is taken as reference and all weight variations are estimated in relation to this reference point. It is argued in a subsequent paragraph that under these conditions, the materials are very near to stoichiometry without oxygen excess or deficiency.

## 3. Results

**Figure 2:**
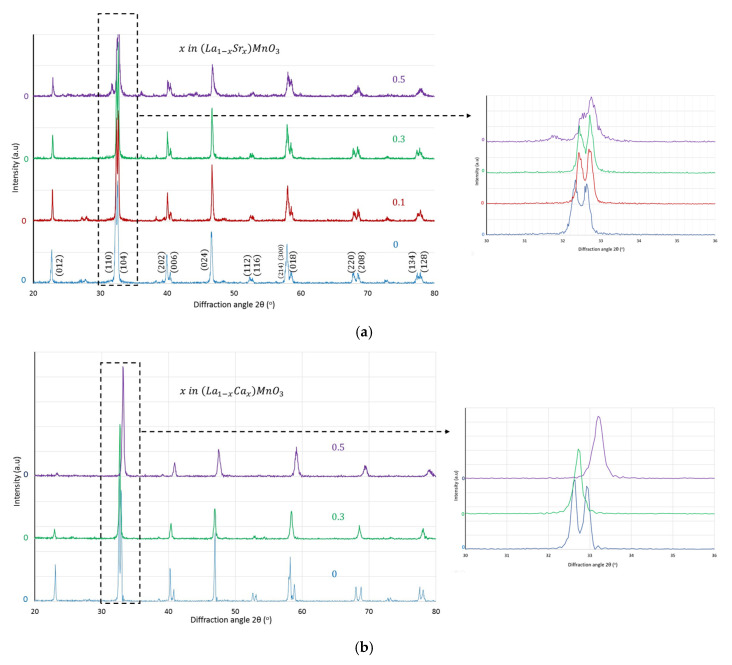
XRD spectra of the as-prepared (**a**) La_1−x_Sr_x_MnO_3_ and (**b**) La_1−x_Ca_x_MnO_3_ materials.

The XRD results of the materials under investigation are shown in Figure 2a,b.

As can be seen, all samples contain only the perovskite phase of the orthorhombic structure. No secondary phases could be detected. Both divalent substitutions Sr^2+^ and Ca^2+^ in La^3+^ positions result in a unit cell contraction since, as indicated in Figure 2, equivalent peaks shift to higher diffraction angles. At first sight, this would appear very strange since the ionic radius of La^3+^ in 12-fold coordination is 1.36 Å which is smaller by ~5.5% than the ionic radius of Sr^2+^ (1.44 Å) and approximately equal to the ionic radius of Ca^2+^ (1.34 Å) in 12-fold coordination [25]. The explanation may probably lie in the nature of the compensating defect for the negatively charged dopant defect $Sr_{La}^{\prime }$ or $Ca_{La}^{\prime }$ (in the following of this article the Kröger–Vink notation will be used for point defect symbols [26]). Positively charged anionic defects or cation interstitials would most probably have resulted in a unit cell expansion. Therefore, the most probable compensating defect is the oxidation of the 6-fold coordinated Mn^3+^ (0.645 Å) to Mn^4+^ (0.53 Å), creating the defect $Mn_{Mn}^{•}$. The accompanied almost 18% reduction is able to accommodate any other expansion effects and leads to a unit cell contraction, as obtained by the XRD results. Conclusively, the XRD results provide a strong indication that the negative charge created by the aliovalent substitutions is compensated by the oxidation of trivalent Mn to tetravalent and not by the creation of oxygen vacancies.

**Figure 3:**
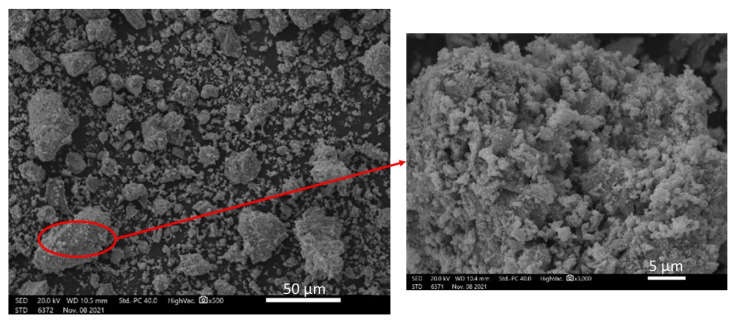
Typical scanning electron microscope photo of the morphology of all synthesized perovskite samples.

A typical scanning electron microscope photo, representative of the morphology of all samples, is shown in Figure 3. As shown, the powders are composed of primary particles in the order of 2–3 μm, the majority of which forms larger porous agglomerates up to 20–30 μm. The specific area of all specimens was between 4–5 m^2^ g^−1^.

### 3.1. La_1−x_M_x_MnO_3_ (M = Sr or Ca): Oxygen Excess Region

**Figure 4:**
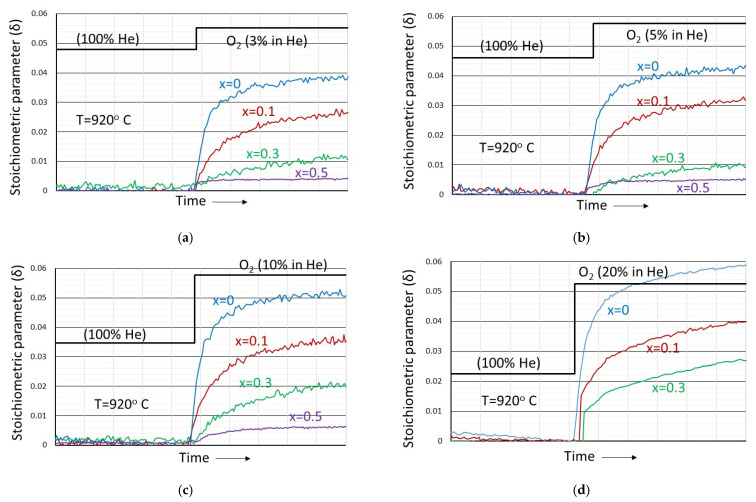
The dependence of the stoichiometric parameter δ in La_1−x_Sr_x_MnO_3+δ_ perovskites initially equilibrated in helium at constant temperature, as a function of x for (**a**) 3% partial pressure of oxygen, (**b**) 5% partial pressure of oxygen, (**c**) 10% partial pressure of oxygen and (**d**) 20% partial pressure of oxygen in helium.

In this experimental series, the La_1−x_Sr_x_MnO_3_ material samples, after being brought at a constant temperature of ~920 °C, are initially left to equilibrate under 100% helium atmosphere until no weight changes are observed. Subsequently, oxygen was introduced in the helium gas stream at partial pressures 3, 5, 10 and 20%, also until no weight changes are observed. The results are shown in Figure 4a–d where the stoichiometric parameter δ (in the formula La_1−x_Sr_x_MnO_3+δ_) is plotted as a function of time. The change in atmosphere is indicated in the figures. In all cases, the reference state is the material in equilibrium with helium where stoichiometry has been assumed (i.e., δ = 0).

As shown in all cases, when in equilibrium with oxygen, the materials take up oxygen and enter into the oxygen excess regime. They are best described by the general formula La_1−x_Sr_x_MnO_3+δ_ which will be further refined in subsequent paragraphs. The oxygen excess, at all other conditions identical, increases with increasing oxygen partial pressure and decreases with increasing Sr^2+^ content. Upon removal of the oxygen, all materials reversibly return to their original stoichiometric state in equilibrium with helium. There is thus an Oxygen Transfer Capacity associated with the ability of the materials to accommodate an excess of oxygen. For LaMnO_3_, which attains the maximum excess under almost atmospheric oxygen partial pressure, this is estimated to be approximately 0.39 wt.%. This is reduced down to 0.04 wt.% for the La_0.5_Sr_0.5_MnO_3_ material that showed the lowest oxygen excess among the tested samples.

**Figure 5:**
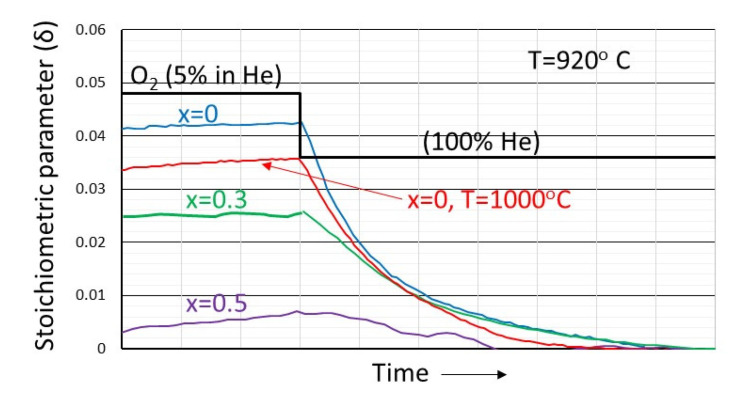
The dependence of the stoichiometric parameter δ in La_1−x_Ca_x_MnO_3+δ_ perovskites as a function of x, initially equilibrated in helium at constant temperature and subsequently subjected to a 100% helium atmosphere.

Similar behavior has been also shown for La_1−x_Ca_x_MnO_3_ materials. The results are shown in Figure 5. The materials after being equilibrated at 920 °C at 5% oxygen are subsequently subjected to a pure helium atmosphere. As can be seen, all weights return reversibly to the weight corresponding in equilibrium with helium. It is worthwhile mentioning that a temperature increase from 920 to 1000 °C resulted in a decreased oxygen excess.

**Figure 6:**
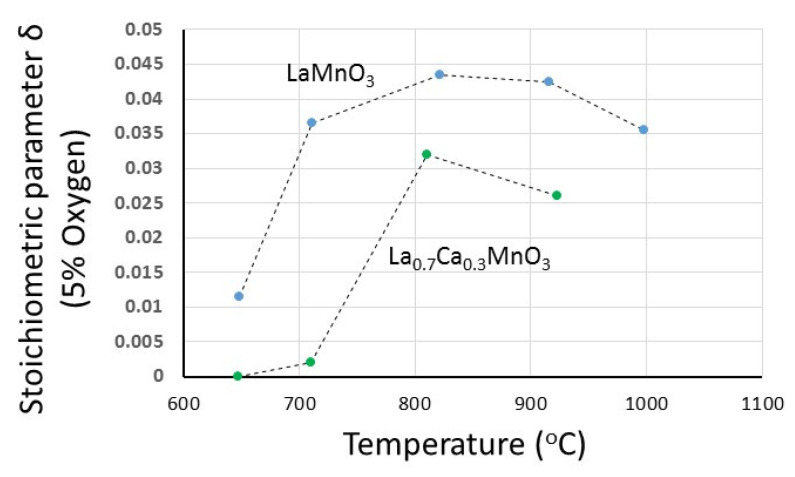
The dependence of oxygen excess (at 5% oxygen) on temperature for LaMnO_3_ and La_0.7_Ca_0.3_MnO_3_.

The temperature effect is quite general and has been observed in both La_1−x_Sr_x_MnO_3+δ_ and La_1−x_Ca_x_MnO_3+δ_ materials. In Figure 6, the dependency of the maximum attainable excess (at 5% oxygen) is shown as a function of temperature for LaMnO_3_ and La_0.7_Ca_0.3_MnO_3_. Similar results have been also achieved with other materials. Above a certain temperature of about 800 °C, the attainable excess decreases as the temperature increases.

### 3.2. La_1−x_M_x_MnO_3_ (M = Sr or Ca): Oxygen-Deficient Region

**Figure 7:**
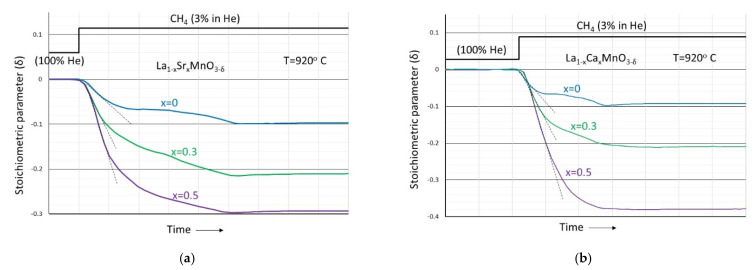
The dependence of the stoichiometric parameter δ in (**a**) La_1−x_Sr_x_MnO_3−δ_ and (**b**) La_1−x_Ca_x_MnO_3−δ_, initially equilibrated in helium and subsequently subjected to a gas mixture of 3% CH_4_ in helium, as a function of divalent ion content x at constant temperature of 920 °C.

In these experimental series, the materials after being equilibrated with helium at a constant temperature of ~920 °C are subjected to a mixture of CH_4_ in helium at a volume percentage of 3%. The weight loss results, converted into the stoichiometric parameter δ, are shown in Figure 7a,b.

**Figure 8:**
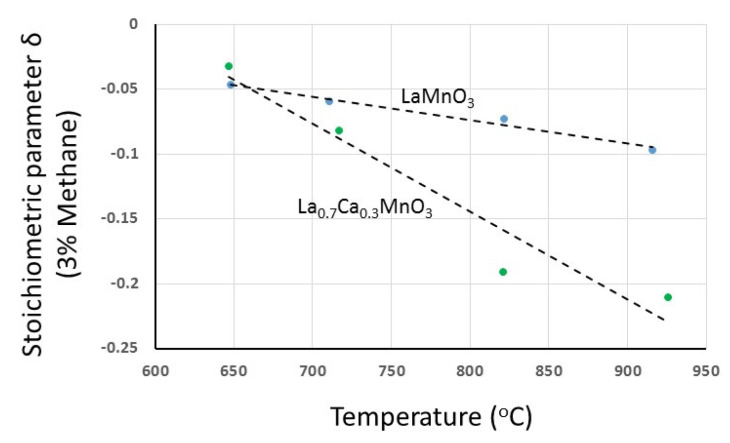
The dependence of oxygen deficiency (at 3% methane) on temperature for LaMnO_3_ and La_0.7_Ca_0.3_MnO_3_.

It is clear that upon the introduction of CH_4_, the materials enter into the anion-deficient region and can be described by the general formula La_1−x_Sr_x_MnO_3−δ_ or La_1−x_Ca_x_MnO_3−δ_. Unlike the oxygen excess region, with all other conditions identical, the deficiency increases with increasing divalent ion content. Upon removal of CH_4_, no weight changes occur, and upon re-introduction of oxygen, all materials recover the weight corresponding to that particular partial pressure of oxygen. The similarities of the deficiency development process during reduction with CH_4_ between the two material families are quite obvious from the figures. In general, two stages can be distinguished: a first initial stage which is characterized by a fast and almost linear rate that increases with increasing divalent ion content (as schematically indicated by the slopes of the dashed lines), followed by a second stage where the rate of oxygen loss takes clearly lower values and declines from the path of a smoothly evolving process running towards completion. As also determined experimentally, the developed oxygen deficiency during reduction with CH_4_ increases with increasing temperature. In Figure 8, the maximum attained deficiency after reduction with CH_4_ is shown as a function of temperature for LaMnO_3_ and La_0.7_Ca_0.3_MnO_3_ materials.

The previously described reversible material reduction is associated with another Oxygen Transfer Capacity which is quite larger than this mentioned in a previous paragraph and was associated with the oxygen excess. For LaMnO_3_, this is estimated to be approximately 0.66 wt.%, while for La_0.5_Sr_0.5_MnO_3_ and La_0.5_Ca_0.5_MnO_3_, it may increase up to 2.2 wt.% and 3.3 wt.%, respectively.

**Figure 9:**
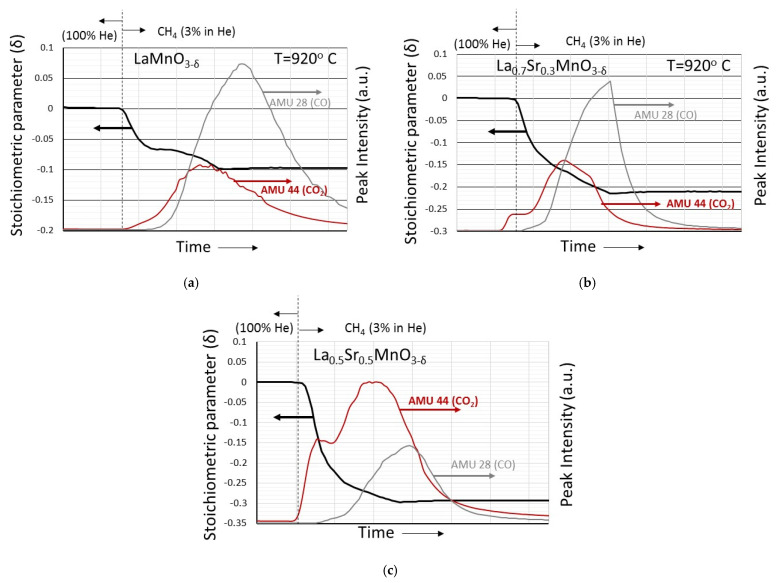
Evolution of CO and CO_2_ characteristic mass spectrometer peaks (at Atomic Mass Units 28 and 44, respectively) that accompany the weight loss curves of (**a**) LaMnO_3−δ_, (**b**) La_0.7_Sr_0.3_MnO_3−δ_ and (**c**) La_0.5_Sr_0.5_MnO_3−δ_ upon reduction with 3% CH_4_ (in He) at 920 °C.

**Figure 10:**
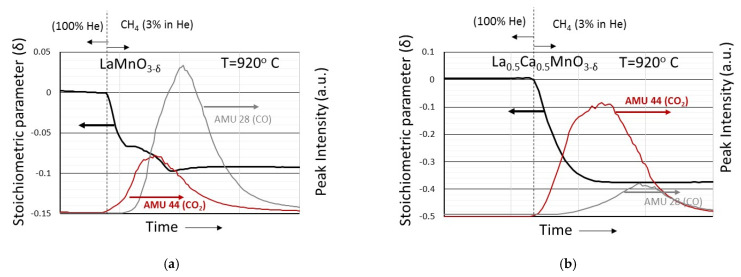
Evolution of CO and CO_2_ characteristic mass spectrometer peaks (at Atomic Mass Units 28 and 44, respectively) that accompany the weight loss curves of (**a**) LaMnO_3−δ_ and (**b**) La_0.5_Ca_0.5_MnO_3−δ_ upon reduction with 3% CH_4_ (in He) at 920 °C.

More information, however, on the CH_4_ reduction process is provided by the mass spectrometer results, shown in Figure 9 and Figure 10, that accompany the weight loss curves. Although qualitative, they contain valuable information on the methane combustion process.

In Figure 9, the CO and CO_2_ mass spectrometer signals (corresponding to Atomic Mass Units 28 and 44, respectively) are shown during the reduction with CH_4_ of samples LaMnO_3−δ_ (Figure 9a), La_0.7_Sr_0.3_MnO_3−δ_ (Figure 9b) and La_0.5_Sr_0.5_MnO_3−δ_ (Figure 9c). In all cases, the evolution of CO_2_ precedes the evolution of CO. For samples La_0.7_Sr_0.3_MnO_3−δ_ and La_0.5_Sr_0.5_MnO_3−δ_, an initial “shoulder” at the CO_2_ peak is distinguishable, the intensity of which increases with increasing divalent cation content. In addition, the intensity of the CO peaks dominates in LaMnO_3−δ_, while the intensity of the CO_2_ peak dominates in La_0.5_Sr_0.5_MnO_3−δ_. Similar characteristics are obtained also for the La_1−x_Ca_x_MnO_3_ samples, as shown in Figure 10a,b. Upon material reduction, initially CO_2_ is evolved and subsequently CO. The CO_2_/CO peak intensity ratio increases as the content of the divalent cation increases. Conclusively, it can be said that during the material reduction process the initial stage is mainly related to methane combustion while the later stage is mainly related to methane reforming. It is worthwhile mentioning that Figure 9a and Figure 10a correspond to the first member of both series, that is actually LaMnO_3_, and provide an impression about the reproducibility of the behavior of the same material made by two different syntheses at different time periods.

## 4. Discussion

### 4.1. LaMnO_3_

It is quite well-accepted that in pure and stoichiometric LaMnO_3_ is the simultaneous presence of Mn^3+^ $\left[Mn_{Mn}^{\times }\right]$, Mn^4+^ $\left[Mn_{Mn}^{•}\right]$ and Mn^2+^ $\left[Mn_{Mn}^{\prime }\right]$. This can be best described by assuming that Mn^3+^ is subjected to a so-called disproportionation equilibrium during which an electron is transferred from one Mn^3+^ to another [27,28]:

(1) $$2Mn_{Mn}^{\times }\overset{{Κ}_{DIS}}{\Leftrightarrow }Mn_{Mn}^{\prime }+Mn_{Mn}^{•}$$

where K*_DIS_* is the equilibrium constant of Equation (1). Assuming that a fraction *ξ* of Mn^3+^ is being disproportionated, the refined chemical formula of the undoped material, written according to the Kröger–Vink symbolism, will be

(2) $$La_{La}^{\times }\left(Mn_{Mn,\;\xi }^{\prime }Μn_{Mn,\;1-2\xi }^{\times }Mn_{Mn,\;\xi }^{•}\right)O_{3}$$

### 4.2. LaMnO_3_ + ΜO (Μ = Sr, Ca)

As indicated by the XRD results discussed earlier, the incorporation of Sr^2+^ or Ca^2+^ as substitutional impurities in La^3+^ positions is most probably compensated by further oxidation of Mn^3+^ to Mn^4+^ [29,30]. For every M^2+^ ion entering the lattice, one Mn^3+^ ion is oxidized. Assuming a content *x* of divalent substitutional ions, the refined chemical formula of the material will be:

(3) $$\underset{A-sites}{\underset{︸}{\left(La_{La,\;1-x}^{\times }M_{La,\;x}^{\prime }\right)}}\underset{B-sites}{\underset{︸}{\left(Mn_{Mn,\;\xi }^{\prime }Μn_{Mn,\;1-2\xi -x}^{\times }Μn_{Mn,\;\xi +x}^{•}\right)}}O_{3}$$

In the broad sense, the material described by (3) can be considered stoichiometric since all oxygen demanded by the cations is present and there is no oxygen excess or deficiency.

### 4.3. Oxygen Excess in LaMnO_3_ + ΜO (Μ = Sr, Ca)

If the stoichiometric material described in (3) attains equilibrium under a certain oxygen (relatively high) partial pressure, then oxygen enters the material which becomes anion excess. Both (La_1−x_Sr_x_)MnO_3_ and (La_1−x_Ca_x_)MnO_3_ materials are well-known to obtain oxygen excess. In a closed packed array of oxygen ions, such as in the perovskite structure, it is energetically very unlikely that oxygen ions will enter into intermediate lattice positions. Most probably, they will create new lattice sites and equal amounts of La^3+^ and Mn^3+^ cation vacancy positions with simultaneous maintenance of the perovskite structure. Here, it has been implicitly assumed that the preferred type of intrinsic point defects are the Schottky defects. Τhis means that three oxygen ions from the atmosphere will enter the structure with the simultaneous creation of Lanthanum and Manganese vacant sites ($V_{La}^{\prime \prime \prime },\;V_{Mn}^{\prime \prime \prime }$) at equal amounts, thus one of each kind. Τhe negative charge of the cation vacancies is compensated by the oxidation of $Mn_{Mn}^{\times }$ to $Mn_{Mn}^{•}$. The equilibrium equation that describes the development of oxygen non-stoichiometry is:

(4) $$6Mn_{Mn}^{\times }+\frac{3}{2}O_{2}\left(g\right)\overset{K_{OX}}{\Leftrightarrow }3O_{O}^{\times }+V_{Mn}^{\prime \prime \prime }+V_{La}^{\prime \prime \prime }+6Mn_{Mn}^{•}$$

where *K_OX_* is the equilibrium constant of Equation (4). According to this mechanism, the oxygen excess materials possess a perfect anion sublattice (no vacancies) and equal amount of cation vacancies on A and B sites. Assuming that *δ* oxygen ions enter per material chemical formula and normalizing (i.e., dividing all subscripts by (3 + *δ*)/3) for a perfect anion sublattice, the refined chemical formula of the material becomes:

(5) $$\left(\underset{A-sites\;\left(sum\;1\right)}{\underset{︸}{{\mathrm{La}}_{La,\;\frac{3\left(1-x\right)}{3+\delta }}^{\times }{Μ}_{La,\;\frac{3x}{3+\delta }}^{\prime }V_{La,\;\frac{\delta }{3+\delta }}^{\prime \prime \prime }}}\right)\left(\underset{B-sites\;\left(sum1\right)}{\underset{︸}{Μn_{Mn,\;\frac{3\xi }{3+\delta }}^{\prime }Μn_{Mn,\;\frac{3\left(1-x-2\xi -2\delta \right)}{3+\delta }}^{\times }Μn_{Mn,\;\frac{3\left(x+\xi +2\delta \right)}{3+\delta }}^{•}V_{Mn,\;\frac{\delta }{3+\delta }}^{\prime \prime \prime }}}\right)O_{O,3}^{\times }$$

Equilibrium reactions (1) and (4) hold simultaneously. Corresponding equilibrium constants are given by the relations:

(6) $$K_{DIS}=\frac{\left(\frac{3\left(x+\xi +2\delta \right)}{3+\delta }\right)\left(\frac{3\xi }{3+\delta }\right)}{{\left(\frac{3(1-x-2\xi -2\delta }{3+\delta }\right)}^{2}}$$

(7) $$K_{OX}=\frac{{\left(\frac{\delta }{3+\delta }\right)}^{2}27\times {\left(\frac{3\left(x+\xi +2\delta \right)}{3+\delta }\right)}^{6}}{{\left(\frac{3(1-x-2\xi -2\delta }{3+\delta }\right)}^{6}}P_{O_{2}}^{-\frac{3}{2}}$$

**Figure 11:**
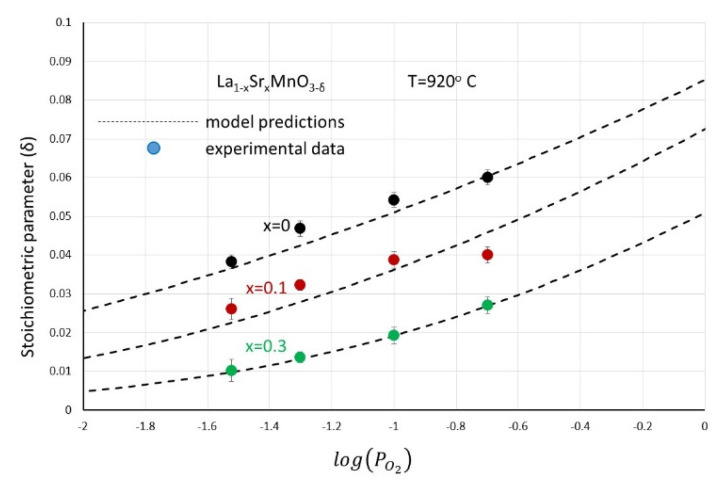
The oxygen excess experimental data of (La_1−x_Sr_x_)MnO_3_ materials (points) in comparison with the predictions of the defect model (dashed lines) expressed by Equations (6) and (7).

By fitting the experimental data of oxygen excess as a function of divalent cation content and oxygen partial pressure reported in Figure 4a–d to the oxygen excess model described above with Equations (6) and (7), Figure 11 is obtained.

As can be concluded from Figure 11, the dependency of the oxygen excess on the divalent ion content and the oxygen partial pressure can be well-explained and are in agreement with model predictions both in the qualitative and quantitative sense. The increased excess with decreasing temperature is also in agreement with the model predictions if one takes into account that reaction (1) is endothermic while reaction (4) is exothermic. The equilibrium constants that resulted from the fitting are *K_OX_* ≈ 3 × 10^−5^ and *K_DIS_* ≈ 9 × 10^−3^ and are in very good agreement with the literature-reported values [31]. In a chemical looping process, this type of reversibly uptaken and removed oxygen is most probably related with a CLOU effect and takes place in gas phase reactions that lead to total combustion [9]. It depends on the partial pressure of oxygen, composition and temperature.

### 4.4. Oxygen Deficiency in LaMnO_3_ + ΜO (Μ = Sr, Ca)

When the stoichiometric material described in (3) is subjected to a CH_4_-containing atmosphere, reduction in the material takes place that liberates oxygen. The most probable and favorable reaction is the reduction of Mn^4+^ [$Mn_{Mn}^{•}]$ to Mn^3+^ $\left[Mn_{Mn}^{\times }\right]\;$with the simultaneous creation of oxygen vacancies $\left[V_{O}^{••}\right]$, according to the equation:

(8) $$O_{O}^{\times }+2Mn_{Mn}^{•}\overset{K_{43RED}}{\Leftrightarrow }2Mn_{Mn}^{\times }+V_{O}^{••}+\frac{1}{2}O_{2}\left(g\right)$$

The higher the content of the divalent cation (x), the higher the concentration of $Mn_{Mn}^{•}$ and consequently the higher the oxygen deficiency that can be attained through Equation (8). This is believed to describe the initial and linear weight loss parts of the curves shown in Figure 7a,b. High Sr^2+^ or Ca^2+^ contents lead to higher Mn^4+^ contents and upon reduction to high oxygen vacancy concentrations. The increased oxygen loss rates that are reflected by the slopes of the dashed lines can be explained if one accepts a reduction rate proportional to the concentration of the reactant $Mn_{Mn}^{•}$. Taking into account the mass spectrometer results shown in Figure 9 and Figure 10 that indicate CO_2_ as the main product, one may conclude that the oxygen liberated through Equation (8) participates in chemical looping combustion. The total CLC reaction can be written as:

(9) $$CH_{4}+4O_{O}^{\times }+8Mn_{Mn}^{•}\overset{}{\to }CO_{2}\left(g\right)+2H_{2}O\;\left(g\right)+8Mn_{Mn}^{\times }+4V_{O}^{••}$$

As long as reaction (9) proceeds to completion and the concentration of $Mn_{Mn}^{•}$ is diminished, a subsequent Μn^3+^ to Mn^2+^ reduction takes place also with simultaneous generation of oxygen vacancies:

(10) $$O_{O}^{\times }+2Mn_{Mn}^{\times }\overset{K_{43RED}}{\Leftrightarrow }2Mn_{Mn}^{\prime }+V_{O}^{••}+\frac{1}{2}O_{2}\left(g\right)$$

This reaction is believed to describe the second weight loss part of the curves shown in Figure 7. According to the mass spectrometry results, the oxygen consumed during this reaction oxidizes CH_4_ partly, resulting in CO and H_2_ as main reaction products. The total chemical looping reforming reaction can be written as:

(11) $$CH_{4}+O_{O}^{\times }+2Mn_{Mn}^{\times }\overset{}{\to }CO\left(g\right)+2H_{2}\left(g\right)+2Mn_{Mn}^{\prime }+V_{O}^{••}$$

### 4.5. Complementary Remarks

Based on the previous discussion, one may expect that the CO_2_ selectivity of La_1−x_Sr_x_MnO_3_ or La_1−x_Ca_x_MnO_3_ should increase with x, as indeed has been reported in the literature [32,33]. The defect model presented in this paper is limited up to x = 0.5. It is believed that this is the border of its validity. It is quite uncertain whether it can describe the whole solid solution series up to x = 1. Nevertheless, the behavior of the end members of the series SrMnO_3_ or CaMnO_3_ are reported to give almost 100% selectivity to CO_2_ [32,33]. This can be understood if one considers that in these materials almost all manganese is Mn^4+^ and the only reduction reaction is reaction (9) that leads to total fuel oxidation. Similar to the effect of Sr^2+^ or Ca^2+^ is the effect of Cu^2+^ substitution on La^3+^ sites, as has been recently reported in the literature [34]. Partial substitution of Mn^3+^ with Al^3+^ leading to the materials La(Mn_1−x_Al_x_)O_3_ is reported to decrease the selectivity to CO_2_ and progressively increase the (already high) selectivity to CO [35]. This can be understood if one considers the manganese disproportionation equilibrium (1) that must be satisfied also in the presence of the dopant. The decrease in the $Mn_{Mn}^{•}$ content, the reduction of which is responsible for CO_2_ production, results in an increase in the CO selectivity. In contradiction, partial substitution of Mn^3+^ with Ni^2+^ leading to the material La(Mn_1−x_Ni_x_)O_3_ is reported to decrease the CO selectivity [36]. This can be understood if one considers $Mn_{Mn}^{•}$ as the compensating defect for $Ni_{Mn}^{\prime }$. Interesting results are presented in [37] where La_1−x_Sr_x_MnO_3_ materials have been investigated for the CH_4_ oxidation under oxygen excess conditions. The most active material appeared to be LaMnO_3_. Under oxygen excess conditions, the materials most probably operate in the oxygen excess region. As outlined in previous paragraphs, the composition that is able to accommodate and deliver the highest oxygen excess is LaMnO_3_.

Finally, during the entire discussion in this paper, the equilibrium with helium has been considered as the reference state. In addition, it has been assumed that the materials at this state are stoichiometric (i.e., δ = 0). This assumption is not totally arbitrary, it is based on the equilibrium δ-logPO_2_ relations published in the literature [29,31]. According to these diagrams, there is a very broad spectrum of oxygen partial pressures extending from ~10^−3^ to 10^−12^ atm where the material is stoichiometric. In the helium gas used, there might possibly be some tenths of ppm oxygen (i.e., PO_2_~10^−4^–10^−5^) which brings, at temperatures between 900–1000 °C, the materials under equilibrium exactly in the stoichiometric region. Even if this was not the case, the oxygen deficiencies at extremely low oxygen partial pressures do not seem to exceed 0.05, which is low compared to the deficiencies achieved when the materials are reduced with CH_4_ (Figure 7). Any possible mistake is therefore considered very small and certainly not able to influence the conclusions derived from this work.

## 5. Conclusions

La_1−x_Sr_x_MnO_3±δ_ and La_1−x_Ca_x_MnO_3±δ_ perovskites exhibit identical defect chemistry and may obtain both oxygen excess and deficiency. The oxygen excess increases as x and temperature decrease, while the oxygen deficiency increases as x and temperature increase. Their Oxygen Transfer Capacity under reductive conditions is composed by two distinct reduction reactions that produce oxygen vacancies: the Mn^4+^→Mn^3+^ reduction drives the CH_4_ oxidation to CO_2_ while the Mn^3+^→Mn^2+^ reduction to CO. The CO_2_/CO is related with the relative contributions of these reactions to the oxygen transfer number and depends on the composition. The selectivity towards CO decreases as x increases. Generally, it can be concluded that the behavior of the materials in a chemical looping process is inherently connected to the defect chemistry model that describes the point defect concentrations.
